# Extracellular Vesicles as Pro- and Anti-inflammatory Mediators, Biomarkers and Potential Therapeutic Agents in Multiple Sclerosis

**DOI:** 10.14336/AD.2021.0513

**Published:** 2021-09-01

**Authors:** Mallahalli. S Manu, Hirohiko Hohjoh, Takashi Yamamura

**Affiliations:** ^1^Department of Immunology, National Institute of Neuroscience, National Center of Neurology and Psychiatry, Kodaira, Tokyo, 187-8502 Japan.; ^2^Department of Molecular Pharmacology, National Institute of Neuroscience, National Center of Neurology and Psychiatry, Kodaira, Tokyo, 187-8502 Japan.

**Keywords:** autoimmunity, extracellular vesicles, Multiple sclerosis, exosomes, microRNA

## Abstract

Multiple sclerosis (MS) is an autoimmune neurodegenerative disease of the central nervous system (CNS) characterized by multiple demyelinating lesions in the spinal cord and brain. Neuronal disruption caused by myelin loss or demyelination, which may accompany axonal changes, leads to multiple neurological symptoms. They may transiently appear for weeks during periods of disease worsening (relapse) in relapsing-remitting form of MS (RRMS). Although a number of genetic, metabolic and environmental factors influencing the development of MS have been identified, the precise mechanisms involved in the CNS tissue damage in MS are still poorly understood. Recent studies have revealed a significant role of circulating extracellular vesicles (EVs) in many diseases. EVs are known to serve as a cellular communication tool between two cell types either in close proximity or in different parts of the body. During the recent development in understanding of the pathogenesis of MS, studies have revealed the possible role of EVs in MS. Furthermore, circulating EVs can be used as a biomarker for monitoring disease progression and activity of MS, and they can also be therapeutic reagents or targets of therapy. In this review we overview and discuss in detail about generation of EVs and their diversified roles in MS.

## 1. Introduction

Multiple sclerosis (MS) is one of the major and common progressive neurological disorders, which affects 2.8 million people around the world in 2020. This number is increased by 20% compared to 2013 data given by Multiple Sclerosis International Federation (MSIF). An alarming increase in the MS numbers in the last two decades is observed in many countries and this rise indicates the necessity and importance of MS research to understand its pathophysiology in order to overcome the suffering.

MS is a devastating disease of the central nervous system (CNS) caused by autoimmune responses to CNS antigens such as myelin basic protein, leading to the neuronal damage and formation of demyelinating plaques in the brain, spinal cord and optic nerves. The immune mediated damage in the CNS of MS patients is caused by complicated interactions among many immune cell types, including T cells, B cells, dendritic cells (DC), macrophages and NK cells [1-3]. Along with immune cells, glial cells such as microglia and astrocytes also act as non-classical immune cells in the pathogenesis of MS [4-7]. CNS damage is caused by immune cells that are activated in the periphery and in the CNS after crossing the blood brain barrier (BBB). They could lead to a variety of symptoms such as fatigue, bladder and bowel dysfunction, visual impairment, movement and coordination problems and sensory disturbances. Patients affected with MS also suffer from cognition and emotional changes which affect the individual living status dramatically [8]. In the past three decades, physicians and researchers have been extensively and collaboratively working in order to clarify the actual cause of MS and its progression. Currently we have substantial evidence to indicate the role of genetic, metabolic and environmental factors that are interconnected to generate hazardous autoimmune responses causing MS [9-13].

Extracellular vesicles (EVs) are the small non-nucleated vesicles derived from various cell types. EVs are usually filled with biomolecules such as proteins, small nucleotide sequence called microRNAs (miRNAs) that are known to serve as regulatory components in several biological responses. Studies conducted during the past two decades have confirmed their role in various human diseases including inflammatory diseases [14]. There is substantial evidence indicating the involvement of EVs in MS pathophysiology, but the functional efficacy of EVs is dependent on their surface molecules and their cargo. Therefore, they can be both beneficiary and harmful. Hence, it has been a great challenge to elucidate their roles in MS disease progression. This review overviews the latest research on EVs, which directly addressed their involvement in MS pathology and their signature in MS progression. Also, we have attempted to consolidate the available information on how EVs from the different sources and cargo can be therapeutically utilized in MS. Lastly, we have discussed the limitation and future prospective of EVs research with significant implication to MS.

## 2. EVs generation and their classification

EVs are the small vesicles mostly of less than 1000 nm in size derived from live cells, whereas apoptotic bodies of a similar size (about 50-5000 nm) are derived from dying (apoptotic) cells. EVs are further classified into microvesicles and exosomes of 100-1000 nm and 50-150 nm in size, respectively. Apoptotic cells often display phosphatidylserine (PS) on their cell membrane, and apoptotic bodies also carry PS on their outer membrane, which may serve as a molecular marker for apoptotic bodies. In contrast, microvesicles are generated by budding from the cell membrane, and selectins, integrins and the CD40 ligand may be used as markers for the microvesicles. Exosomes, on the other hand, are formed by inward budding of the membrane of multivesicular endosomes. Thus, endosomes contain large quantities of endosome-associated proteins such as Rab GTPase SNAREs (soluble N-ethylmaleimide-sensitive fusion protein attachment protein receptors), Annexins and flotillin. In addition, CD63, CD81 and CD9 that belong to a membrane-spanning protein family are abundant in exosomes and can be used as markers for exosomes. In addition, heat shock proteins (HSP60, HSP70, HSP90), major histocompatibility complex (MHC) class I and II antigens and nucleic acids (DNA and RNA) are also contained.

Exosomes as well as microvesicles are involved in maintenance and regulation of vital functions of cells via cell-to-cell communication between cells in long-distance or between adjacent cells. In the immune system, for instance, it has been reported that exosomes released from T cells may modulate antigen-presentation functions of the antigen presenting cells by transferring miRNAs in the exosomes, by which immune responses are regulated [15]. On the other hand, regarding the mechanism of cancer cell metastasis, exosomes released from breast cancer have been shown to be capable of altering BBB, thereby enhancing the brain metastasis [16].

In the history of research for EVs, an important discovery was that they carry nucleic acids such as messenger RNAs (mRNAs) and miRNAs [17]. RNAs from donor cells may be selectively incorporated into EVs and delivered to recipient cells as messages from donor cells. Compared to mRNAs encoding polypeptide sequences, miRNAs are small non-coding RNAs of 21-23 nucleotides in length. miRNAs are incorporated into the RNA-induced silencing complex (RISC) and function as mediators in gene silencing. In this process, translation of mRNAs having partial complementarity to the miRNA is inhibited, whereas mRNAs carrying nearly complement sequences to the miRNA are digested [18, 19]. As such, miRNAs play important roles in gene regulation via suppression of their target genes in cells. Thousands of miRNA genes have been found in animals and plants, and expression profiling of miRNAs has revealed their tissue- and developmental stage-specific expression and also disease-associated expression[20-24]. Thus, miRNAs are closely related to various vital functions and pathogenesis of diseases via gene silencing, implying that they may serve as useful biomarkers or targets of therapy in human diseases.

Cells of different origin and characters produce EVs that contain different RNA repertoires. For instance, the exosomes from T, B, and dendritic immune cells are identified to contain miRNA repertoires characteristic for each cell population (15). Changes in EVs may represent altered homeostasis and become a useful marker for monitoring life maintenance systems and diseases.

## 3. EVs released by immune cells

The immune system is one of the complex and highly regulated systems in the human body. Almost every organ has its own immune protection system utilizing immune receptors, which are used to transmit external signals. Several communication modes like cell-cell interaction, cytokine signaling, and enzymatic reactions are known to be involved in the immune regulation. Researchers in the last two decades have shed light on the role of EVs in regulation of immune responses in normal and disease conditions [25-27].

Most of the immune cells can secrete EVs that control cellular response. For example, DCs secrete EVs at different stages of cellular activation, thereby regulating diverse functions of target cells. In fact, the large EVs of immature DCs induce T cell activation and also promote secretion of Th2 cytokines from the target T cells, whereas small EVs from the same cells induce Th1 cytokines. Both large and small EVs are capable of inducing IFN-γ secretion from Th lymphocytes [28]. Another study showed that EVs derived from DCs contain cell surface molecules like MHC, ICAM-1(Intercellular Adhesion Molecule 1) and some other costimulatory molecules that can promote an efficient induction of the T cell activation [29, 30].

EVs derived from T cells can also modulate the function of recipient cells. Exosomes/ectosomes derived from T cells may control the endothelial cells gene expression in CD47 dependent manner [31]. Recent studies showed that T cell-derived EVs controls B-cell responses and also have the potential to control the germline center reaction and antibody production [32, 33]. Key regulatory cells like Foxp3^+^ regulatory T cells (Tregs) also secrete EVs, which may regulate the production of IL-10 and IL-6 from DCs by utilizing miRNAs contained in the EVs [34]. Therefore, functions of EVs released from immune cells may depend on the physiological environment and types of cellular events.

## 4. Immune response and MS pathology

CNS inflammation is the primary damage taking place in the pathology of MS. Formation of demyelinating plaque in the CNS, which is caused by inflammatory processes, is a pathological hallmark of MS [35]. It is thought that autoreactive T cells, which recognize a CNS-derived peptide bound to MHC class II molecules, play a central role in the formation of CNS lesions. The T cells are activated in the periphery, then migrate to the CNS and produce inflammatory cytokines to damage the myelin and oligodendrocytes [36]. More recently, drugs targeting T cell migration have shown efficacy in MS, indicating the involvement of T cells in MS pathophysiology. Experimental autoimmune encephalomyelitis (EAE), a mouse model for human MS, is immensely used to explore the mechanism and pathophysiology of MS [37]. Experiments using EAE models have greatly helped the researchers to elucidate the roles of individual immune cells in the pathogenesis of MS and to identify potential targets of therapy or therapeutic strategies. T cells specific for a peptide capable of inducing EAE (such as myelin oligodendrocyte glycoprotein (MOG) 35-55) play a major role in EAE and are called encephalitogenic T cells. Encephalitogenic T cells contain Th1 cells which secrete IFN-γ and Th17 cells producing IL-17. Activated encephalitogenic T cells can pass through the BBB, reach the CNS and are reactivated in response to antigens presented by local antigen presenting cells, leading to the secretion of more cytokines to attract other immune cells like B cells, macrophages, and DCs. To some extent, these cells can also activate microglia and astrocytes in the CNS, which significantly contribute to inflammatory reactions [38-42].

## 5. The role of EVs in MS

EVs have diverse roles and broad targets, and individual EV appears to have its own mode of action, according to their origin. MS is the CNS autoimmune disease involving multiple inflammatory processes, which take place in the periphery, BBB and CNS parenchyma. Complex interactions between many immune cell types are found to occur in each process. EVs originated by immune cells may promote the progression of MS, but it is possible that they would also suppress the disease in a distinct manner[43]. Because of the versatility of EVs, we will discuss the various roles of EVs in MS at different subheadings.

### 5.1 EVs as pro-inflammatory regulator in MS

**Figure 1.**
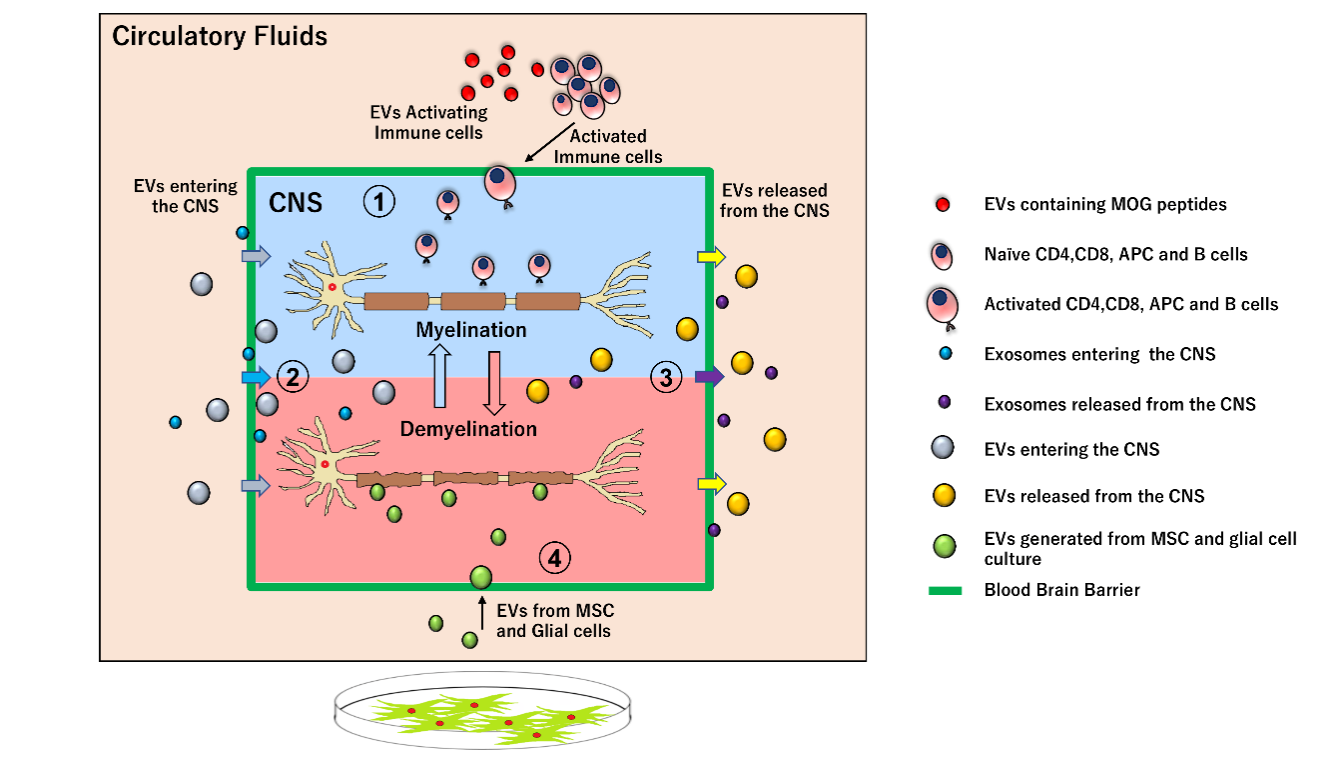
**Role of EVs in Multiple sclerosis**. (1) EVs act as proinflammatory regulator. EVs generated by immune cells in the periphery acts as proinflammatory signals for the activation of naïve immune cells that migrates and disrupt the CNS tissue. (2) EVs entering the CNS. EVs generated in periphery can cross the BBB and reaches the CNS tissue during inflammatory reaction. (3) EVs released from the CNS. EVs generated in CNS during inflammatory reaction will be released into the circulation. (4) EVs from Mesenchymal stem cells and Glial cells. EVs generated from MSC and Glial cell culture can reach the CNS inflammatory site and helps in remyelination of neurons.

Exosomes play a potent role as a proinflammatory regulator in autoimmune diseases such as rheumatoid arthritis, Graves’ disease and MS [44-46]. The previous study indicated that microparticles from RRMS patients act together with inflammatory mediator thrombin to disrupt the endothelial barrier function, and the cargo inside the EVs may act as a proinflammatory signal in many circumstances [47-49]. In the EVs transfer experiments in mice model of MS, circulating plasma EVs of EAE mice induces the spontaneous relapse-remitting phenotype in MOG immunized mice, although this mouse model usually develops monophasic disease. Moreover, they showed that fibrinogen present in the transferred plasma EVs drives the CD8+ T cells which may contribute to induction of spontaneous EAE in transferred mice [50, 51]. In the study, B cells derived from MS patients were found to generate exosome enrichment factors with different protein content after *in vitro* culture. Interestingly, these factors have the potential to induce oligodendrocyte death but not death of microglia or astrocytes [52]. Our group has recently showed that exosomal miRNA in the plasma samples of MS patients can target regulatory T cells and disrupt the immune regulatory mechanism. In the study, we identified that miRNAs in the exosomes from RRMS are significantly altered as compared to exosomes from healthy subjects. In particular, an increase of miRNA let-7i was notable. Transferring the exosomes enriched with let-7i from MS patients to the naïve CD4+ T cells inhibit the differentiation of T reg cells [53]. These studies have indicated that EVs have the potential to regulate inflammatory responses in MS (Fig. 1(1)).

### 5.2 EVs as biomarker in MS

EVs are considered to provide potential biomarkers for screening or monitoring of many diseases. Numerous efforts were made to evaluate the role of EVs as a biomarker for MS. In this regard, EVs content and surface markers are the major target in the past studies. Microparticles-concentration was found to greatly increase in plasma and cerebrospinal fluid (CSF) of MS patients, and then efforts were shifted to identify the phenotype of the EVs, enabling to track the possible generator of EVs. EVs containing surface CD31^+^ microparticles thought to be derived from endothelial cells of BBB, plasma platelet microparticles from circulating platelets, and CD4^+^/CCR3^+^ and CD4^+^/CCR5^+^ ones from the activated T cells were identified in plasma and CSF samples derived from MS patients by independent groups [54-57]. In the samples from MS patients, there were also EVs containing myelin proteins like MBP (myelin basic protein), PLP (proteolipid protein), and MOG. Presence of these proteins was confirmed in exosomes derived from serum, CSF, and peripheral blood mononuclear cells. The amount of MOG in the exosome strongly correlated with disease activity [58]. Particle size distribution of plasma EVs in MS patients differs from that of healthy individuals, and mass spectroscopy analysis of small EVs from the MS patients has showed increased C16:0 sulfatide (sulfatide with short chain fatty acid) that can be a biomarker for MS [59]. Another study was carried out to evaluate differential surface protein expression in MS. Interestingly, serum EVs from RRMS showed the increased TLR4 and decreased TLR3 receptor expression compared with healthy ones [60].

Welton and colleagues studied the differences in the EVs content in the CSF of MS patients, and revealed that EVs isolated from CSF of RRMS patients express more Plasma kallikrein and Apolipoprotein-E4 compared to those from healthy individuals. Functional enrichment analysis has also revealed a strong association of 50 upregulated proteins with biological processes that are associated with MS pathology [61]. In another study, up-regulation in the sphingomyelin pathway has been observed in the CSF from MS, which was associated with generation of more ceramide in the CSF. Interestingly, there were higher numbers of acid sphingomyelinase-enriched exosomes in the CSF of MS, which well correlated to enzymatic activity and to disease severity of MS [62]. Although several groups have tried to identify an EV biomarker relevant for MS, such efforts have not been successful. We speculate that circulating EVs could be generated and released by CNS cells. But, it is also possible that EVs released from the peripheral tissue may cross the BBB and enter the CNS tissue (Fig. 1(2, 3)). Assuming this, it seems to be rewarding to pursue efforts to seek for a biomarker useful for diagnosis and management of MS (Table 1).

**Table 1:** EVs as a biomarker in MS.

Disease Progression	EVs Marker	Source	Reference
RRMS/SPMS	CD4^+^/CCR3^+ and^ CD4^+^/CCR5^+^	CSF	(54)
CIS/RRMS/SPMS	CD31+ Endothelial microparticles	Plasma	(55, 56)
RRMS	plasma platelet microparticles	Plasma	(57)
RRMS/SPMS	myelin basic protein, proteolipid protein, and myelin oligodendrocyte glycoprotein	Plasma CSF PBMC	(58)
SPMS	C16:0 sulfatide	Plasma	(59)
RRMS	TLR4	Serum	(60)
RRMS	kallikrein and Apolipoprotein-E4	CSF	(61)
RRMS/CIS/PCP	acid sphingomyelinase	CSF	(62)

### 5.3 Exosomes (Small EVs) in MS

Exosomes are the class of small EVs released by various cell types, and has a wide variety of application in biology [63, 64]. Exosomes derived from the CNS cells could be released into blood stream and circulate throughout the body. *In vitro* experiments have also indicated that they have potentials to cross the BBB [65, 66]. Most of the EVs are enriched with small RNAs, possessing the ability to control a variety of physiological processes. Exosome-associated miRNAs are one of the major candidates that can control expression of mRNA and can be the biomarker for human diseases. Studies of serum samples from MS patients showed that several exosomal miRNAs are differentially expressed, depending on disease activity and progression status. Techniques such as microarrays, next generation sequencing, and real time expression analysis have contributed to profiling the exosomal miRNAs. Several groups including ours have already reported differentially expressed EVs miRNA in MS. As described above, Kimura and collogue have shown the up-regulation of miR-let-7i, miR-19b, miR-25, and miR-92a in circulating exosomes in patients with RRMS. Of the miRNAs, the expression of miR-let7i was increased in MS, and moreover, the miRNA can potentially suppress the immune regulation mediated by Treg cells. Mechanistically, miR-let7i suppresses the generation of Treg by targeting insulin like growth factor 1 receptor (IGF1R) and transforming growth factor beta receptor 1 (TGFBR1). Consistently, IGF1R and TGFBR1 expressed on circulating naive CD4^+^ T cells is reduced in patients with MS [67].

In another study, miR-15b-5p, miR-451a, miR-30b-5p, miR-342-3p were shown to be dysregulated in RRMS patients, whereas miR-127-3p, miR-370-3p, miR-409-3p, miR-432-5p were dysregulated in patients with secondary progressive MS (SPMS) as compared with healthy controls. When the miRNA from EVs of the two different stages of MS, namely RRMS and SPMS, was compared, miR-15b-5p, miR-23a-3p, miR-223-3p, miR-374a-5p, miR-30b-5p, miR-433-3p, miR-485-3p, miR-342-3p, and miR-432-5p were greatly dysregulated [68]. In the study by Selmaj et al., RRMS patients showed up-regulated miR-196b-5p, miR-301a-3p, and miR-532-5p and down-regulated miR-122-5p, miR-196b-5p, miR-301a-3p, and miR-532-5 in circulating plasma exosomes [69]. The othre study on CSF samples showed that MS patients have increased amounts of miR-146a-5p, miR-181a and miR-223 [70] (Table 2).

In summary, small EVs are filled with various contents that have different roles individually in the progression of disease, which can modulate the gene expression in the target cells. Therefore, increases of small EVs in MS may have a direct impact on pathogenesis (Fig. 1(2.3)).

**Table 2:** Small EVs (Exosomes) and their microRNA content in MS.

Disease Progression	Dysregulated miRNA	Derived from	Reference
RRMS/SPMS vs HC	Up regulated miR-let-7i, miR-19b, miR-25, miR-92a	Circulating exosomes	(67)
RRMS vs HC	miR-15b-5p, miR-451a, miR-30b-5p, miR-342-3p	Circulating exosomes	(68)
SPMS vs HC	miR-127-3p, miR-370-3p, miR-409-3p, miR-432-5p		
RRMS vs SPMS	miR-15b-5p, miR-23a-3p, miR-223-3p, miR-374a-5p, miR-30b-5p, miR-433-3p, miR-485-3p, miR-342-3p, miR-432-5p		
RRMS vs HC	Up regulated miR-196b-5p, miR-301a-3p and miR-532-5p	Circulating exosomes	(69)
	Down regulated miR-122-5p, miR-196b-5p, miR-301a-3p, and miR-532-5		
MS vs HC	Up regulated miR-146a-5p, miR-181a and miR-223	CSF exosomes	(70)

### 5.4 EVs in MS therapy

In another approach for research, EVs are regarded as therapeutic targets or reagents in treating human diseases, including MS [71]. Some recent studies have shown that EVs derived from mesenchymal stem cells (MSCs) of adipose tissue would promote recovery from demyelination in an animal model for progressive MS. Notably, transfer of MSCs has been evaluated for years as cell therapy for MS. As the outcome of transferring EVs from MSC, they observed microglial polarization, up-regulation of CD4^+^CD25^+^FOXP3^+^ regulatory T cells (Treg), and a reduction in plasma proinflammatory cytokines. *In vitro* analysis showed that exosomes derived from MSCs stimulated by IFN-γ are able to suppress T cell proliferation, and Th1 and Th17 cytokine production including IL-6, IL-12p70, IL-17AF, and IL-22 in an *in vitro* culture, though they increased the levels of indoleamine 2,3-dioxygenase, a known anti-inflammatory enzyme. In addition, MSC-exosomal derived miRNA can control the microglial polarization towards M1 or M2 phenotype along with altering cytokine profiles [72-75].

EVs can be engineered to carry desired cargo and to deliver it at specific target site, Casella et al. recently developed murine microglial cell line that produces EVs that express endogenous “eat me” signal Lactadherin (Mfg-e8) on the surface and are filled with IL-4. A single injection of the engineered EV reduced neuro-inflammation in the mice induced for EAE, and the EAE clinical score was reduced associated with up-regulation of anti-inflammatory markers in phagocytic cells [76]. Recently the same group have shown that, naturally derived EVs from oligodendrocytes culture contains myelin antigens and transfer of this EVs reduce EAE pathophysiology in a myelin antigen-dependent manner in EAE models [77]. Interestingly EVs derived from MSC protects oligodendrocytes from DNA damage and EVs from MSC co-cultured with microglia helps the myelin repair by recruiting oligodendrocyte precursor (OPC) in demyelinated regions[78, 79].

It is widely known that relapse frequency of MS is markedly reduced in pregnant women with MS [80]. Williams and his colleague have looked into the involvement of EVs in EAE model and reported that levels of circulating exosomes are increased during pregnancy and an injection of the exosomes into EAE mice suppresses T cell activation and helps the maturation and migration of oligodendrocyte precursor cells (OPC) into active lesions [81]. Taken together, EVs generated from MSC, microglia and oligodendrocytes have a potential to treat demyelination lesions or restoring immune tolerance in MS (Fig. 1(4)) (Table 3). Even though EVs have shown a promising result in treatment of MS, there are some lacunas before completely understanding their therapeutic, biological and functional properties. Further comprehensive research is required to make EVs as therapeutic agents in MS treatment.

### 5.5 EVs as a therapeutic biomarker in MS

Fingolimod is a first-in-class orally bioavailable drug used to treat MS. Fingolimod in the phosphorylated form binds to the lymphocytes S1P1 receptor with high-affinity, thus preventing egress of lymphocytes from the secondary lymphoid organs and entry of pathogenic T cells into the CNS of MS patients [82]. Analysis of fingolimod-treated MS patients showed dramatic changes in the concentration of EVs and their miRNA content with altering immune regulatory activity within 5 h after fingolimod administration [83]. Another challenge in treatment of MS is for many patients with MS who are not responsive to available drugs [84]. In this view, Manna et al. has shown interesting results, indicating that two exosome-associated miRNAs expression might be utilized in identifying IFN-β response groups. In relation with this, miR-22-3p and miR-660-5p were significantly up-regulated and about 14 miRNAs were downregulated in IFN- β - treated RRMS patients with response to therapy compared to those without response [85]. Therefore, in future there are possibilities of adopting EVs evaluation as standard biomarker for prediction or early decision for the drug efficacy in individual MS patients.

**Table 3:** EVs in MS therapy.

EVs origin	Target	Result	Reference
MSCs from human adipose tissue	Reduced glial fibrillary acidic protein and Iba-1 in brain	Diminishing brain atrophy and promoting remyelination	(72)
MSCs stimulated by IFNγ	Reduced IL-6, IL-12p70, IL-17AF, and IL-22	Upregulates regulatory T cells numbers	(73)
Bone marrow MSCs	Increases in IL-10, TGF-β, Decreases TNF-α, IL-12	Regulating the polarization of microglia	(74, 75)
Microglia	upregulating anti-inflammatory markers in phagocytic cells	Reduced Neuroinflammation	(76)
Oligodendrocytes	Induce immunosuppressive monocytes and apoptosis of autoreactive CD4+ T cells	Reduced EAE pathophysiology	(77)
Placenta-derived Mesenchymal stem/stromal cells	Reduced DNA damage in oligodendroglia populations	Increased myelination in Spinal cord	(78)
Microglia co-cultured with MSCs	Promoted oligodendrocyte precursor cells recruitment	Myelin repair	(79)

## 6.0 Limitations and Future prospective

Since most of the cells shed the EVs in normal physiological process, EVs are widely distributed throughout the body fluids. Therefore, categorization of EVs based on their origin, targets or function is still a challenge. Although EVs research requires detailed and deep studies to confirm their role in disease pathophysiology, MS is an autoimmune disorder influenced by multiple genetic and environmental factors, which would hamper the research into the role of EVs in MS. The past analysis showed that EVs from MS patients’ blood samples contains proteins and miRNA`s targeting BBB and regulatory T cells differentiation. Nevertheless, it is obvious that more concrete evidence is required to establish their role in MS pathophysiology. Several research groups have tried to correlate the characters of circulating EVs in MS patients with those in EAE model mouse, with a purpose to utilize the EVs as a signature biomarker in MS screening. Unfortunately, there was nonconsistency in the results, possibly due to the influence of environmental and genetic factors. However, more robust and detailed studies on EVs surface molecules and their cargo considering several influencing factors may help to identify the candidate signature in MS disease activity and progression.

Because of the circulating property, EVs has been considered as a future therapeutic delivery system. In fact, *in vivo* injections of EVs generated from MSC of several tissue types and healthy glial cells like microglia and oligodendrocytes have remarkably reduced the inflammatory reactions in EAE models. Also, efforts to generate engineered EVs with desired peptide cargo to target pathogenic cells were successful and proved to be efficacious in prevention of EAE model. Given such promising data, we believe that more efforts on this direction to relay on EVs as a therapeutic agent will be rewarding for future treatments of MS.

## 7.0 Conclusion

The cause and pathogenesis of MS is still not clear, but several efforts to stop the progression of MS with drugs resulted in making significant progress. Given the circulating properties of EVs along with the surface bound receptors and cargo contents, EVs may have a significant role in the immune cell signaling involved in MS progression. Because of the potentiality of EVs contents like miRNAs to regulate mRNA expression, EVs may become a potential biomarker in diagnosing and monitoring the disease progression and also can be a therapeutic target in MS treatment. Since their regulatory mechanisms would be worked in several levels in a complicated manner, more extensive studies to analyze EVs carefully and to elucidate their role in connection to MS pathogenesis and treatment are needed in the future.
